# Insane Asylums

**Published:** 1854-05

**Authors:** 


### INSANE ASYLUMS.

Insanity and the provision for the unhappy beings who are afflicted with disease of the mind, are subjects which have derived fresh interest from events just transpiring in Congress. A bill, it is known, recently passed both Houses of our National Legislature ceding to the States a large amount of the public lands, for the benefit of the indigent insane, and it is also now known that the President has vetoed that bill. It is a matter of the most sincere regret, we would say, of the deepest grief, that the President has felt constrained to defeat so beneficent a measure. Most of the States, it is true, have erected Asylums for the Insane, but in none is the provision equal to the wants of the population. Amply as some have prepared for such sufferers, there is not a State in the Union which has all the accommodations necessary for its insane, and in most of the States the provision is lamentably inadequate. There are still many lunatics confined in the common jails because there is not room for them in the Lunatic Asylums. In view of these facts the regret, we think, must be general, that the humane law passed by Congress was not permitted to go into operation.

Lunatic Asylums having in view the restoration of the insane are institutions of a modern date. As early as 1553 there was one in England, where the crazy were kept, not with a thought of their cure, but for the security of the public. PINEL first conceived the benevolent idea of restoring them to reason, and since his day they have been treated as the subjects of bodily disease, and with what success is well known to every one. Nothing in the history of modern medicine is more honorable to it than the signal improvement in the management of mental alienation. Multitudes of insane men are every year restored to the use of their faculties, to their families, and to the world. It is done through the agency of these institutions, for no fact is now considered better established than that it is necessary to remove deranged persons from their families, and bring them under the restraints and the curative influences of a Lunatic Asylum.

We subjoin an extract from the *New York Times* which contains some excellent thoughts on this subject forcibly expressed:—

"The insane behave better when together,—when kept under established rules to which they readily submit, since they are not arbitrarily imposed upon single individuals,—where they may be occupied in work or sports as is deemed best,—where schools, reunions, music may employ some hours, and seclusion afford perfect quiet at appointed intervals. In PINEL's day it was estimated that of every hundred of the insane, from twenty to thirty were violent; now, not over five or six are so in a hundred. Before Asylums were common, the insane were shut up in private chambers from the beginning to the end of the year, immured in poorhouses, or chained in prisons. Their shrieks, sounding through the quiet of a rural village, chilled the blood of cheerful youth, and frightened into gloomy melancholy all that heard them. Infectious cells, dungeons unfit for the condemned, were their dreary life-graves, till exhausted nature left them tamer and fitter for the company of drivelling idiots, in disgusting almshouses. Now, provision is made for their care—and if the malady is not of too long-standing, for their cure. Yet, no State in the Union provides enough for all her insane, nor does any one provide, perfectly, for those it has in charge. To deal with them as they should be dealt with, is an exceedingly delicate matter. Attendants must be firm and unyielding, yet gentle and winning. Medical assistants must be shrewd—gifted in reading human nature—the choice ones of their profession. Asylums should avoid all that seems prison-like in architecture or furnishing. Rooms for restraint should be strong, without seeming so; the bolts should be out of sight, and the locks sure, without pretension. The parlors should be hung with pictures suggestive of quiet and domesticity. Apartments to confine the noisy, while not far removed from the centre, yet should be constructed with thick walls, to deaden the sound that none need be shocked by their noise, nor the sleepers disturbed. None but those who know the insane, their wants, their dangers, their difficulties, should be intrusted with the plans for building asylums. In these respects, much yet remains to be done. The failure of the favorite project of Miss Dix must not lessen the hopes of philanthropists, or slacken their efforts. May a different effect be the issue, and greater interest than ever be felt in all that concerns the welfare of those in whom reason is overthrown.

